# Circulating MicroRNAs as Potential Biomarkers of Exercise Response

**DOI:** 10.3390/ijms17101553

**Published:** 2016-10-05

**Authors:** Mája Polakovičová, Peter Musil, Eugen Laczo, Dušan Hamar, Ján Kyselovič

**Affiliations:** 1 Hamar Institute for Human Performance, Faculty of Physical Education and Sports, Comenius University Bratislava, Nábr. Arm. Gen. L. Svobodu 9, Bratislava 814 69, Slovakia; 2 Department of Pharmacology and Toxicology, Faculty of Pharmacy, Comenius University Bratislava, Odbojárov 10, Bratislava 832 32, Slovakia; peter.musil@uniba.sk (P.M.); kyselovic@uniba.sk (J.K.); 3 Department of Track & Field, Faculty of Physical Education and Sports, Comenius University Bratislava, Nábr. Arm. Gen. L. Svobodu 9, Bratislava 814 69, Slovakia; eugen.laczo@uniba.sk; 4 Department of Sport Kinanthropology, Faculty of Physical Education and Sports, Comenius University Bratislava, Nábr. Arm. Gen. L. Svobodu 9, Bratislava 814 69, Slovakia; dusan.hamar@uniba.sk

**Keywords:** circulating microRNA, biomarker, physical exercise, skeletal muscle

## Abstract

Systematic physical activity increases physical fitness and exercise capacity that lead to the improvement of health status and athletic performance. Considerable effort is devoted to identifying new biomarkers capable of evaluating exercise performance capacity and progress in training, early detection of overtraining, and monitoring health-related adaptation changes. Recent advances in OMICS technologies have opened new opportunities in the detection of genetic, epigenetic and transcriptomic biomarkers. Very promising are mainly small non-coding microRNAs (miRNAs). miRNAs post-transcriptionally regulate gene expression by binding to mRNA and causing its degradation or inhibiting translation. A growing body of evidence suggests that miRNAs affect many processes and play a crucial role not only in cell differentiation, proliferation and apoptosis, but also affect extracellular matrix composition and maintaining processes of homeostasis. A number of studies have shown changes in distribution profiles of circulating miRNAs (c-miRNAs) associated with various diseases and disorders as well as in samples taken under physiological conditions such as pregnancy or physical exercise. This overview aims to summarize the current knowledge related to the response of blood c-miRNAs profiles to different modes of exercise and to highlight their potential application as a novel class of biomarkers of physical performance capacity and training adaptation.

## 1. Introduction

Athletic performance depends on a number of physiological, mental and environmental factors closely related to the training level, nutritional status and genetic predisposition of an athlete. Physical activity and exercise training induce changes in extracellular and intracellular signaling that influence the expression of genes controlling inflammation, angiogenesis, mitochondrial synthesis, myocardial and skeletal muscle metabolism, regeneration and remodeling [1,2,3,4]. 

Gene transcription and translation are the starting phases of the human body’s adaptation to exercise which determine changes in levels and activity of many proteins and hormones associated with the physical performance [5]. In the early state of the research, RNA has been viewed as a simple working copy of the genomic DNA, transporting information from the genome into the proteins. Since the turn of the millennium numerous studies changed this rather simple concept and revealed that a large fraction of the genome sequences increases RNA transcripts that do not code proteins, called non-coding RNAs (ncRNAs) [6]. In recent years, it has become increasingly apparent that ncRNAs, although located in the non-protein coding parts of the genome called genomic wastelands, are of crucial importance for the normal development and function of the human organism [7]. 

Among ncRNAs, the identification of microRNAs (miRNAs) in the early 1990s opened a new level of complexity in transcriptional and translational regulation [8]. Mammalian miRNAs have been identified as regulators of gene expression by repressing specific target genes at the post-transcriptional level. miRNAs have proven to be critical mediators of the response to cellular stress related to disease and environmental stimuli [9]. In mammals, highly conserved miRNAs are predicted to regulate, by post-transcriptional or translational mechanisms, the expression of about 50% of the protein-coding genes [10]. The precise mechanism of miRNAs targeting, as well as its activity, is still not fully understood. A single gene can be regulated by multiple miRNAs, and likewise, a single miRNA may regulate more target genes that are often grouped in a specific biological pathway [11]. To date, over 2500 miRNAs have already been identified in humans [12]. Similar to other regulatory molecules, the expression of miRNAs frequently changes due to a disease. Most of the miRNAs identified to date have been associated with cancer, cardiovascular diseases, diabetes, inflammation, and neurological disorders [13,14,15,16,17]. Important roles of miRNAs have emerged in the control of metabolic pathways involved in lipid and glucose metabolism [18], energy homeostasis and nutrition [19]. Evidence is also growing that miRNAs play a role in governing cell senescence and a panel of aging-associated miRNAs was identified [20].

miRNAs genes have the ability to control cell proliferation and apoptosis and are located at fragile sites in the genome regions. Their deregulation may significantly contribute to proliferative diseases such as cancer [21,22]. Due to the pivotal role of miRNAs in disease development, they have been subjects of intensive research either as drug targets or diagnostic markers. Some of them have already achieved an application in clinics. MRX34 (Mirna Therapeutics Inc., Austin, TX, USA), miRNA-34 tumor suppressor entered clinical testing in 2013 and it is now in open-label phase I clinical trial in liver cancer patients [23]. The first human miRNA-based therapeutic, Miravirsen (Santaris Pharma-Roche Innovation Center Copenhagen, Copenhagen, Denmark), inhibitor of miRNA-122 biogenesis has initiated the phase III of clinical testing for hepatitis C virus infection treatment [24]. In the area of cancer clinical diagnostics several tests using miRNAs as biomarkers are already available. miRNA expression profiles can be used to precisely classify various types of cancers, and are superior in the classification of poorly differentiated tumors. Molecular diagnostics company Rosetta Genomics Ltd. (Rehovot, Israel). offers a miRNA panel to identify unknown primary origin of metastatic cancers [25,26]. miRNAs’ diagnostic assay from Prometheus Laboratories Inc. (San Diego, CA, USA) allows to identify the origin of metastatic cancer with further classification of 25 particular tumor types [27]. 

An interesting feature of miRNA activity is that while miRNAs are often moderate regulators under homeostatic conditions, their function becomes more amplified in response to injury or excessive stress [28]. miRNAs have been identified as intracellular modulators of mitochondrial metabolism, inflammation, muscle recovery and hypertrophy. These findings attracted the attention of sports scientists and started the research on miRNAs regulation in exercise physiology [29]. The identification of miRNAs expression pattern characterizing physical exercise could be applied for monitoring of physical fatigue and recovery and even to evaluate physical performance capacity [30]. Moreover, as circulating miRNAs (c-miRNAs) are of tissue or cellular origin, some studies investigated miRNAs as potential markers of doping manipulations. Recently, a panel of distinct plasma miRNAs for the detection of autologous blood transfusion doping has been successfully specified and is currently considered to be used as auxiliary parameters in World Anti-Doping Agency (WADA) Athlete Biological Passport concept [31].

## 2. MicroRNA Biogenesis and Function

**Figure 1:**
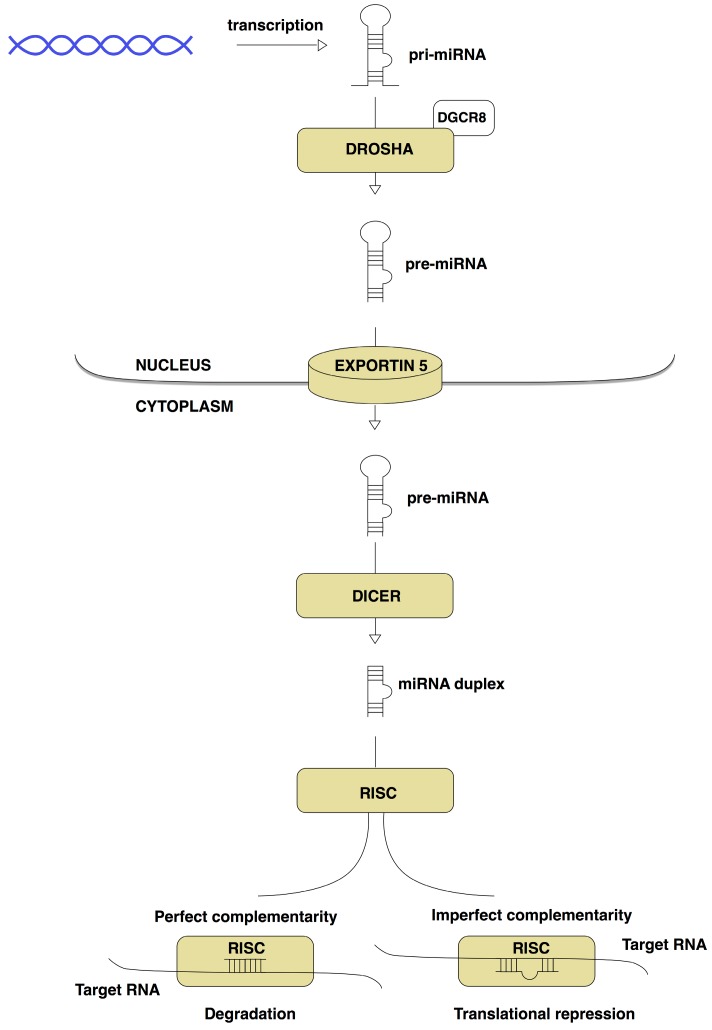
Biogenesis of miRNA. miRNAs genes are transcribed as long primary transcripts primary microRNA (pri-miRNA), which are subsequently processed by RNA polymerase II Drosha resulting in precursor miRNA (pre-miRNA) hairpins with a length of about 70 nucleotides. Pre-miRNAs are exported into the cytoplasm through the Exportin 5 protein and then cleaved by the Dicer complex to imperfect miRNA-miRNA* duplexes about 22 nucleotides in length. miRNA duplexes are separated to form the mature miRNAs and short single stranded miRNAs are incorporated into the RNA-induced silencing complex (RISC) causing the translational repression or mRNA degradation.

The biogenesis of miRNAs (Figure 1) is a multistep process beginning in the cell nucleus, where miRNA encoding genes are transcribed and generate precursors. miRNA genes are evolutionary conserved and are located within the introns or exons of protein coding genes, as well as in intergenic areas. They are transcribed in the same way as protein coding genes [32]. Initially, a primary miRNA (pri-miRNA) double-stranded transcript with several hundred base pairs is formed in the nucleus, usually by the enzyme endoribonuclease (RNase) II. Afterwards, pri-miRNA is processed by nuclear RNase III enzyme Drosha and its cofactor DiGeorge Syndrome Critical Region 8 (DGCR8), called Pasha, into a 60–70 nucleotide long precursor miRNA (pre-miRNA) hairpin [33]. Pre-miRNA is then exported to the cytoplasm by Exportin 5 protein where it is subsequently processed by the Dicer, RNase III into the 19–24 nucleotide long mature miRNA duplex [34]. Finally, the double-stranded mature miRNA is unwound and the single active strand, also known as a guide strand, is loaded into the RNA-induced silencing complex (RISC) containing endonuclease Argonaute-2 along with other proteins and causing the degradation of mRNA [35]. The second passenger strand is usually degraded, although there are some reports emerging that the passenger strand could be active in gene regulation as well. However, miRNA biogenesis also involves other mechanisms via non-canonical Dicer or the Argonaute-independent pathway [36].

Depending on the recognition site, the binding of RISC to the target site in mRNA can have different effects. In the case that the target site is perfectly complementary to the miRNA, the target mRNA is cleaved by RISC [34]. Binding to partially complementary sites in mRNA is typical in mammals and results in repression of translation or degradation of the target transcript [37,38]. 

Nucleotides 2–8 of the miRNA are particularly important for pairing with the target mRNA. This sequence motif is referred to as the miRNA seed sequence and is often a primary determinant for target recognition. Since most target sites on mRNA have only partial base complementarity with their matching miRNAs, individual miRNAs may target as many as 100 different mRNAs [11]. Likewise, individual mRNA may comprise multiple binding sites for different miRNAs resulting in a complex regulatory network. Considering this mode of action, it is possible that the vast majority of cellular and extracellular functions are regulated by miRNAs either directly or indirectly [39].

Individual miRNA species are assigned a numeric name in sequential order according to the date of discovery and classification. Orthologous or identical miRNA sequences found in multiple species are assigned the same numeric value with a species-specific prefix. Many miRNAs are clustered into the families based on the similarities of the sequence in the seed region capable of targeting the common sets of genes [40].

Although some miRNAs are tissue-specific, most miRNAs show a broader tissue distribution. Tissue-specific miRNAs are defined as mature miRNAs which are expressed 20-fold higher in a specific tissue than the mean levels in all other tissues [21]. Up to now, no tissue or organ lacking miRNAs expression has been identified. miRNAs fine-tune gene expression and have a big impact on gene regulation nodes. 

## 3. Skeletal-Muscle Specific miRNAs

Skeletal muscle is a highly plastic organ able to alter its phenotype in response to external stimuli such as neuromuscular activity, mechanical load and nutrition. Myogenesis and muscle metabolism are controlled by several signaling pathways regulated by a broad spectrum of miRNAs. Many of them are broadly expressed across many cell types, whilst some are expressed in a tissue-specific manner. Both categories play an important role in muscle tissue development and function [41]. The miRNAs most abundant in muscle tissue, commonly referred to as myomiRs, operate as modulators of skeletal and cardiac muscle proliferation, differentiation, metabolism and hypertrophy. The myomiR family includes miR-1, miR-133a, miR-133b, miR-206, miR-208a, miR-208b, miR-486 and miR-499 [42]. Most myomiRs are expressed both in the heart and skeletal muscle, except for miR-208a, which is cardio-specific, and miR-206, which is skeletal muscle-specific [43]. As individual miRNAs can act upon numerous target mRNAs and every mRNA can be targeted by multiple miRNAs, identification of biologically important target genes remains a major challenge in miRNA research. Despite this complex problem, numerous biological targets for muscle specific miRNAs have been identified or computationally predicted. A comprehensive overview of myomiRs and their molecular targets was published by Horak et al. [44].

### 3.1. MyomiRs Regulation in Skeletal Muscle 

The significant role of myomiRs in the regulation of skeletal muscle functions has been manifested in various stages of muscle maintenance. Skeletal muscle cell development is driven by proliferation of myoblasts and subsequent differentiation into multinuclear myotubes. During muscle cell differentiation the largest changes occur in miR-1, miR-133a, miR-133b, miR-206, miR-486 and miR-499 expression. MyomiRs miR-1, miR-206 and miR-486 promote myoblast differentiation and miR-133a enhances myoblast proliferation [45,46]. 

MyomiRs have proven to directly target pathways regulating skeletal muscle hypertrophy and participate in the regulation of muscle cell characteristics related to fiber types [47,48]. Beta-myosine heavy chain gene expression responsible for the slow-twitch type I muscle fibers development showed a correlation with increased levels of miR-206, -208b and miR-499. It is likely that the ratio of miR-206, miR-208b and miR-499 expression may help to distinguish between fast-twitch and slow-twitch skeletal muscle phenotypes [49]. 

Regeneration process of muscle fibers was linked with enriched distribution level of miR-206, which could regulate a retrograde signaling pathway required for nerve-muscle interactions and may serve as an indicator of motor innervation [50,51]. MyomiRs miR-1 and miR-206 were proven to be upregulated in satellite cells after injury, promoting muscle regeneration by affecting Pax 7 [52].

Deregulation of myomiRs is established across many human muscular disorders [53]. A clinical trial showed that miR-1, miR-133, and miR-206 were able to discriminate patients affected by Duchenne as well as Becker’s muscular dystrophy from healthy controls [54]. A study of miR-206 expression detected that the upregulation of miR-206 coincides with the progression of amyotrophic lateral sclerosis [55]. The downregulation of miR-1, miR-133a, miR-133b and miR-206 expression in tissues of rhabdomyosarcoma tumors was observed comparing to healthy skeletal muscle tissue [56,57]. Reduced levels of miR-1 were correlated with worsened muscle function in patients with chronic obstructive pulmonary disease [58]. The impact of immobilisation on myomiRs expression was observed in a study on healthy young men. Seven days of bed rest resulted in the downregulation of miR-1 and miR-133a [59]. 

### 3.2. Modulation of miRNA Expression in Skeletal Muscle Tissue by Exercise

Since skeletal muscle is a highly plastic tissue, able to alter its phenotype in response to various neuromuscular activity, several studies investigated changes in myomiRs expression modulated by exercise of varying intensity and mode, using muscle biopsy technics. 

The first study demonstrating that myomiRs expression in human skeletal muscle responds to the exercise was the one of Nielsen et al. [60]. The authors were assessing the levels of myomiRs expression in samples obtained by skeletal muscle biopsy from the vastus lateralis after an acute bout of endurance exercise. They found significantly increased level of miR-1 and miR-133, however only before, but not after the 12-week endurance training program. In addition, the levels of miR-1, miR-133a, miR-133b and miR-206 identified in the resting period after the systematic training were downregulated comparing to the levels before initiating the endurance training trial. Russel et al. revealed that an acute bout of endurance cycling exercise upregulated miR-1, miR-133a and miR-133b, whilst the endurance training lasting 10 days led to the upregulation of miR-1 and downregulation of miR-133b [61]. The regulation of skeletal muscle miRNAs associated with resistance training was observed in a study which involved 56 young untrained men performing a five-day-per-week resistance training program for 12 weeks [62]. A series of 21 miRNAs were detected from vastus lateralis muscle biopsies. Distribution patterns of miR-26a, miR-29a, miR-378 and miR-451 were related to changes in functional hypertrophy and, moreover, were able to distinguish between low and high responders to resistance training. Interestingly, the expression levels of myomiRs were unaffected after this particular resistance training program. In elderly participants, the strength training lasting 12 weeks resulted in downregulation of miR-1 [63]. miRNAs altered by muscle atrophy may play a role in the age-related loss of skeletal muscle [64].

Results of contemporary research prove that disease, injury, exercise or inactivity can alter miRNAs expression pattern, including myomiRs, and are supposed to play a decisive role in phenotypic changes. Until recently, the miRNA expression profiles stem from tissue samples was obtained by invasive muscle biopsy technics, entailing considerable discomfort for the study subjects. The discovery that a large amount of tissue-specific miRNAs exists in plasma, serum and other biofluids has led to intensive investigation and identification of c-miRNA signatures, which can be used as fingerprints of various physiological and pathological conditions. The identification of c-miRNAs expression pattern, specifically regulated by various modes of exercise, could reveal unique biomarkers of exercise physiology and could provide further insight into the molecular control of exercise adaptation.

## 4. Circulating miRNAs

The majority of miRNA have been found intracellularly. However, many reports in the last decade have confirmed that miRNAs enter the circulation system including blood and other body fluids [65]. Despite the accumulating evidence of miRNAs in body fluids, the origin and function of c-miRNAs remains poorly understood [66]. It is not clear if miRNAs are actively secreted to the circulation and have an endocrine function, or whether they are just passively leaking following cell damage. Some studies suggested that miRNAs are involved in cell communication, indicating that c-miRNAs, like hormones and cytokines, may act as mediators of gene expression in the target cells [67,68,69,70]. 

Several research papers have concluded that c-miRNAs are not only quite stable in most body fluids, but remain stable even under harsh conditions that can degrade most RNAs, such as extreme pH levels, long-term storage at room temperature, and repetitive freezing and thawing [71,72,73]. 

Most of the c-miRNAs are protected from RNase degradation in the blood by lipid vesicles such as exosomes, [74] microparticles, [75] and apoptotic bodies [76] or by association with RNA-binding protein such as Argonaute-2 [77] or high-density lipoproteins [78]. 

The presence of miRNAs in microparticles has led to the hypothesis of a selective miRNA export system and the possibility of circulating miRNAs to operate in cell-to-cell communication [68]. An alternative theory supposes that c-miRNAs can be merely by-products of cellular activity or damage and persist in the circulation due to the high stability of miRNAs-protein complexes [69,77,78]. The findings of miRNAs in circulation also raise a question whether c-miRNA concentrations match their tissue expression levels. So far, only a few studies, mostly on oncology patients, have addressed this issue and the findings have been rather inconclusive. Some researchers described similar alteration trends for both circulating and tissue miRNAs [79], whereas others reported that only a subset of c-miRNAs reflect tissue expression profiles [80]. Despite the question of their origin, the identification of multiple miRNA changes in circulation can provide a useful reference related to a particular physiological or pathological state. Although c-miRNAs have been successfully identified as biomarkers for a number of diseases, the most widely reported are studies of c-miRNAs as cancer biomarkers [81,82,83,84,85,86]. 

For the detection and quantification of miRNA expression profiles in body fluids, several approaches are available, such as miRNA microarrays, methods of quantitative real time Polymerase Chain Reaction (qPCR) and next generation sequencing [87]. The most widely used are microarrays and qPCR methods. Microarrays make it possible to achieve high throughput screening and are usually used for the discovery and identification of c-miRNA biomarkers. They tend to have lower sensitivity and dynamic range. On the other hand, qPCR provides the advantage of high specificity and sensitivity and it is considered as the gold standard for c-miRNA detection in clinical laboratories, despite suffering from a lack of throughput issues. Being easily accessible and routinely processed in medical assessment, plasma and serum represent the most promising and extensively studied sources of c-miRNAs [88]. Recently, reports on using the whole blood samples for c-miRNA analysis were published, which may be considered as an attractive and easy handling specimen. Due to the relative convenience of extraction, quantification, detection and progress in detection technology, c-miRNAs have become attractive non-invasive biomarker candidates in comparison with other molecules such as proteins [89]. 

## 5. The Effect of Different Modes of Physical Exercise on Circulating MicroRNAs Profile

Extracellular c-miRNAs that are responsive to exercise stimuli have received attention as potential biomarkers of physical fitness, performance potential and training adaptation. Physical exercise and training induce numerous physiological alterations in cardiovascular system, angiogenesis, cell metabolism, inflammation and muscle remodeling. To date, 17 studies investigating the influence of physical exercise on c-miRNAs levels in plasma and serum have been published [90,91,92,93,94,95,96,97,98,99,100,101,102,103,104,105,106]. 

### 5.1. Circulating miRNAs Plasma or Serum Profiles Altered by Exercise

An overview of altered c-miRNAs expression profiles in blood serum or plasma by various exercise types is presented in Table 1.

The research in circulating miRNAs responsive to exercise is very recent and the number of studies is limited. Presented results are sometimes contradictory and divergent. Most of the findings reported were based on relatively small samples with inconsistent methodologies and study designs. Another major source of inaccuracy and variability in the data from different laboratories is the absence of a commonly accepted experimental protocol and reference genes for data normalization in c-miRNA quantification.

The majority of published studies were focused on the influence of endurance exercise on the expression pattern of c-miRNAs [90,91,92,93,94,95,96,97,98,99,100]. Only four studies have investigated the impact of strength training on c-miRNA levels [102,103,104,105]. Aerobic endurance and strength or resistance type of exercise represent opposite ends of the adaptation process. Endurance exercise activates a variety of metabolic and morphological changes such as mitochondrial biogenesis, muscle fast-to-slow twitch fibre-type transformation and substrate metabolism. Strength training stimulates muscle hypertrophy and increases protein synthesis and maximal contractile force output. Each mode of exercise results in activation of specific signalling pathways and subsets of genes transcriptionally regulated and fine-tuned by microRNAs [4]. 

The pioneering study investigating c-miRNA expression profiles in relation to physical exercise was published by Baggish et al. in 2011 [90]. The study examined the plasma profiles of specific c-miRNAs involved in angiogenesis, inflammation, hypoxia/ischemia, skeletal and cardiac muscle contractility. The c-miRNAs expression profiles were measured at rest and after an acute exhaustive cycling exercise in a cohort of competitive male rowers, before and after a sustained period of aerobic training. They observed three distinct c-miRNAs expression patterns responsive to exercise. A linear correlation was observed between the expression level of miR-146 and the aerobic performance parameter VO_2max_. 

The influence of marathon running on c-miRNAs expression has been observed in seven studies [91,92,93,94,95,96,101]. Detected myomiRs were upregulated in all of the studies except one focused on inflammatory c-miRNAs [94]. 

Global changes in miRNA expression in response to exercise were investigated in two studies [97,98]. Nielsen et al. performed 724 miRNAs screening measurements and quantified the expressions of 188 c-miRNAs in plasma [97]. Results of this study demonstrated that acute endurance training robustly modifies miRNA expression patterns in plasma. Authors hypothesized that the increase in myomiRs observed is due to the selective secretion rather than passive release caused by muscle damage.

In the subgroup of a longitudinal cohort trial of aerobic fitness Nord-Trøndelag Health Study 3 (HUNT3), the screening study of 720 miRNAs expression profiles in serum before the treadmill test were explored [98]. Results indicate that levels of miR-210, miR-21 and miR-222 could be used to distinguish between low and high VO_2max_ responders and possible utilization of miR-210 as a biomarker of aerobic fitness. 

Aoi et al. found that c-miR-486 level is significantly decreased after the acute bout and chronic aerobic exercise and is negatively correlated with VO_2max_ [99].

Only recently, the study investigating whether high-intensity interval exercise is superior to vigorous-intensity continuous exercise was reported. Response of c-miRNAs on these two regimes of exercise revealed the same expression pattern of detected miRNAs [100].

Studies evaluating circulating miRNAs expression response to the resistance exercise are less reported. The comprehensive microarray analysis of c-miRNAs in serum after a bout of acute resistance exercise was published by Sawada et al. [103]. Unexpectedly, the microarray analysis of miRNA expression profile revealed no changes in muscle specific miRNAs in circulation.

Contrarily, the study of c-miRNA expression responsive to five months of resistance training in a small cohort of older adults revealed altered profiles of myomiRs, both in muscle tissue and blood plasma [104]. The plasma and muscle miR-499 abundance was identified as the most sensitive marker of the increase in knee extensor strength with resistance training. 

A trial including an untrained control group was reported by Wardle et al. [105]. They investigated whether levels of c-miRNAs differ between endurance-trained and strength-trained cohorts of elite male athletes. Plasma levels of miR-21, miR-221, miR-222 and miR-146a were significantly higher in endurance athletes than in strength athletes.

Plasma levels of c-miRNA altered by acute and chronic aerobic exercise were investigated in chronic kidney disease patients. Acute cycle-ergometer exercise upregulated miR-125b in chronic kidney disease patients only, and miR-150 in both patients and healthy control group. Downregulation of miR-146a after the acute exercise was observed in kidney disease patients only. 12 weeks of home-based aerobic training led to the downregulation of miR-210 after the acute exercise bout in chronic kidney patients group only, not in healthy subjects [106].

Due to the diverse nature of study designs, the number and type of detected c-miRNAs responsive to exercise summarized in Table 1 vary significantly. However, some c-miRNAs listed in Table 2 indicate differential expression pattern depending on type and duration of the exercise.

miR-21 differentially responding to exercise in circulation is well-known oncomiR that affects tumor-developing pathways such as sustained proliferation through PTEN (Phosphatase and Tensin Homolog), Sprouty, PI3K (Phosphoinositide 3-Kinase), PDCD4 (Programmed Cell Death Protein 4), impaired apoptosis through *BTG2* (B-cell Translocation Gene 2), FasL (Pro-apoptic FAS Ligand), FBXO11 (F-box Protein 11), and TIMP3 (Tissue Inhibitor of Metalloproteinases 3) [107]. Other targets of miR-21 are associated with immunity and the pathogenesis of autoimmune diseases such as SPRY1 (Sprouty RTK Signalling Antagonist 1), GNAQ (Guanine Nucleotide-Binding Protein Alpha-Q), PLEKHA1 (Pleckstrin Homology Domain Containing Family A), and CXCR4 (C-X-C Chemokine Receptor 4) [108]. In addition, it has been reported that miR-21 regulates adipogenic differentiation through the modulation of TGF-β (Transforming Growth Factor Beta) signalling and promotes renal fibrosis in diabetic nephropathy by targeting PTEN (Phosphatase and Tensin Homolog) and SMAD7 (Mothers Against Decapentaplegic Homolog 7) [109].

The next differentially expressed miR-146a plays a key role in the innate immune response and adaptive immune response by targeting TRAF6 (Tumor Necrosis Factor Receptor Associated Factor 6), IRAK1 (Interleukin 1 Receptor Associated Kinase 1) and IL-8 (Interleukin 8) mRNAs. Additional miR-146a targets have been recently reported. In vascular smooth muscle cells miR-146a targets KLF4 (Kruppel-Like Factor 4). New target CXCR4 (C-X-C Chemokine Receptor 4) was identified in leukemic cell lines and in normal megakaryocytes [110].

Molecular targets of miR-148/152 family members are associated with cell motility or/and cell growth. MiR-148a has many different targets such as PXR (Pregnane X Receptor), CAND1 (Cullin-Associated and Neddylation-Dissociated 1), HLA-C (Human Leukocyte Antigen C), ACVR1 (Activin A Receptor Type 1), IGF-IR (Insulin-Like Growth Factor-1 Receptor), IRS-1 (Insulin Receptor Substrate 1), CCK2R (Cholecystokinin-2 Receptor) and PTEN (Phosphatase and Tensin Homolog) [111].

The cluster miR-221/222 targets various kinds of genes and molecular pathways. Experimentally verified targets include *BBC3* (BCL2 Binding Component 3), *BMF* (Bcl2-Modifying Factor), *CDKN1B* (Cyclin-Dependent Kinase Inhibitor 1B), *CDKN1C* (Cyclin-Dependent Kinase Inhibitor 1C), *ESR1* (Estrogen Receptor 1), *FOXO3* (Forkhead Box O3), *ICAM1* (Intercellular Adhesion Molecule 1), *PTEN* (Phosphatase and Tensin Homolog) and many others [112]. miR-221 and miR-222 play a remarkable role in vascular biology and vascular pathology [113]. In different muscle lineage cells, miR-221 and miR-222 activate regenerative processes through targeting key cell cycle regulators p27 and p57, involved in the induction of expression of contractile proteins [114]. miR-222 is a negative regulator of adipocyte insulin sensitivity in humans and rodents and seems to be a potential biomarker of metabolic diseases [115]. 

The last miRNA exhibited sensitive expression to mode of exercise is let-7d. It belongs to the Let-7 family that is well-conserved across species with a substantial role in developmental processes. It seems to be involved in the regulation of glucose metabolism by targeting INSR (Insulin Receptor) and IRS-2 (Insulin Receptor Substrate 2) in skeletal muscle [115]. The group of genes regulated by let-7d includes also genes such as *CDC25A* (Cell Division Cycle 25 Homolog A), *CDK6* (Cyclin-Dependent Kinase 6), *C-MYC* (V-Myc Avian Myelocytomatosis Viral Oncogene Homolog), *KRAS* (Kirsten Rat Sarcoma Viral Oncogene Homolog), *HMGA2* (High Mobility Group AT-Hook 2) and *IMP-1* (Insulin Like Growth Factor 2 MRNA Binding Protein) [116]. 

### 5.2. Circulating miRNAs Whole Blood Profiles Altered by Exercise

Eight studies [117,118,119,120,121,122,123,124,125] have evaluated c-miRNAs responsive to exercise in the whole blood samples. Tonevitsky et al. presented a complex study of dynamically regulated miRNA-mRNA networks by cardiorespiratory exercise screened for 20,000 mRNAs and 200 miRNAs in the whole blood samples. Samples were collected at four time points during the 30 min of running on treadmill at 80% VO_2max_. They identified 298 differentially expressed mRNAs and 5 miRNAs [117]. 

In a study of changes in the leukocyte methylome after a sprint interval training, the downregulation of miR-21 and miR-210 was found [118]. 

The influence of cycling ergometer exercise on the expression level of c-miRNAs in neutrophils was investigated by Radom-Aizik et al. [119]. They have found that brief aerobic exercise changes the expression pattern of 38 c-miRNAs out of a total 826 screened miRNAs, and 3 biochemical pathways linked to inflammation have been identified as being altered by exercise.

The same laboratory investigated the impact of brief aerobic cycling ergometer exercise on c-miRNA panel in peripheral blood mononuclear cells. They identified 34 miRNAs altered by exercise, most of which regulates inflammatory processes. Comparing miRNAs patterns responsive to the same type of exercise authors declared only a little overlap between altered c-miRNAs in peripheral blood mononuclear cells and monocytes [120].

A similar type of brief cycling ergometer exercise was applied to study the changes of c-miRNAs expression profile in monocytes. Exercise led to the altered expression of 19 c-miRNAs that could be involved in attenuation of pathological activation of monocytes and vascular health [121].

An interesting study of the impact of exhaustive stepwise exercise test on miRNA expression patterns characteristic for various human diseases in peripheral blood was investigated in a group of elite endurance athletes and moderately active controls. The observed changes in 24 different miRNAs profiles have been shown to be insignificant and support the idea that disease-associated miRNAs are not readily altered by physiological exercise and therefore, they are useful as disease biomarkers [122]. This inference was modified in a very recent study where c-miRNAs variation between endurance and strength athletes in plasma and whole-blood samples was tested. Authors observed 231 c-miRNAs in plasma/serum and 265 c-mirRNAs in the whole blood samples differentially expressed after the 6 days of endurance or strength training. The effect sizes of differences in blood outreach the differences in plasma samples. They investigated the association of exercise altered c-miRNAs with different diseases and pathologies and proven a high correlation of miRNAs regulated by training with miRNAs signatures affected by diseases [123].

Even more recently, a study investigating the impact of exercise on angiogenesis associated c-miRNAs in children was published [124]. High-volume cycling exercise increased miR-16 and miR-126 levels, whereas high-intensity cycling showed no influence on circulating levels of studied miRNAs. 

Physical activity, as a well-known factor combating aging process, has been positively correlated with longer leukocyte telomere length [126,127]. Impact of acute treadmill running exercise on the expression of telomeric genes and miRNAs levels in white blood cells was studied by Chilton et al. [125]. They detected 56 miRNAs differentially regulated post-exercise. miR-186, miR-181, miR-15a and miR-96 were significantly upregulated 60 min after the exercise and in silico analysis indicated telomeric genes as their potential targets. 

## 6. Discussion

Since their discovery, miRNAs have shown many promising perspectives in a wide range of clinical applications, not only for diagnostic purposes, but in therapy as well. A huge number of studies have been focused on their potential as biomarkers for various diseases, particularly for cancer. In a similar manner, physical exercise has been shown to be an activator of gene expression and miRNA levels that vary considerably depending on the mode of exercise [30,59,60,61,62,63,90,91,92,93,94,95,96,97,98,99,100,101,102,103,104,105,106,117,118,119,120,121,122,123,124,125]. Many studies revealed miRNAs to have a potential as markers of muscle function and exercise adaptation. Physical exercise modulates the function of many physiological systems in the human body. The effect of exercise is dependent mainly on its type, intensity and duration. The adaptations to the endurance exercise elicit muscle-based and systemic responses including an improvement of metabolic, neuromuscular and contractile functions in muscle, a decrease in glycogen storage, an increase in mitochondrial biogenesis, and the modulation of oxidative stress, systemic inflammation and immune responses. Responses to resistance training occur primarily in the neurological, muscular and endocrine systems [128]. The usage of miRNAs as markers of exercise response remained limited due to the necessity of using invasive muscle biopsy procedure for obtaining samples. This problem has been substantially alleviated by recent techniques for the detection and identification of stable miRNAs in body fluids. Less demanding access to the specimen has opened a new door for more extensive miRNA biomarkers research.

This field is in its infancy and the number of studies is limited by inconsistent study designs, exercise protocols, analytical detection and small number of participants. The majority of published studies were oriented on the influence of the endurance exercise on the c-miRNAs expression. However, also a few verified changes of c-miRNAs responsive to resistance training were reported. We focused on circulating profiles in plasma or serum, because the number of studies analyzing the whole blood was small and specimens heterogeneous. The magnitude of observed changes in c-myomiRs expression across the studies was not possible to assess due to the inconsistency of analytical protocols. myomiRs were included in the investigation in most of the studies. miR-1, miR-133 and miR-206 levels were upregulated in all of the studies with the exception of those in which the analytical threshold of detection could not be reached or myomiRs were not included in the consideration [91,92,93,95,96,97,100,104]. miR-499 was upregulated in three studies, one of them was chronic resistance training [104] and two of them were marathon runs [91,99]. Cluster of miR-208a,b was upregulated after the endurance aerobic exercise. The level of miR-208b was increased after the strenuous [92] and acute eccentric exercise [102] (Table 1). One possible hypothesis explaining the unique upregulation of c-myomiRs regardless of exercise type could be the overlap in gene targets between myomiR family members [43,44]. Upregulation of miR-208a was observed after the marathon run, a strenuous endurance type of exercise [91]. Because miR-208a is the heart-specific miRNA, it is supposed to be minimally affected by non-cardiac tissue injury. This observed expression could be explained in the context of extremely demanding physical load or with the role of the heart system in the regulation of systemic metabolism with the miR-208a as a component of metabolic regulation [129]. Focused on non-muscle specific miRNAs, upregulation of angiogenic miR-126 [130] was associated with four aerobic exercises with different intensities and duration [91,101].

Six c-miRNAs, miR-21, miR-146-a, miR-148a, miR-221, miR-222 and let-7d (Table 2) showed differential expression depending on various mode of exercise. They regulate a broad spectrum of genes and nowadays it is not possible to identify its origin in circulation. However, all of them regulate key pathways in angiogenesis and inflammation [107,108,109,110,111,112,113,114,115,116]. Differential response of these c-miRNAs in serum or plasma may reflect adaptation to specific exercise regime, as their expression varied depending on endurance versus strength training and acute versus chronic stimulation.

The studies of altered c-miRNAs by exercise in the whole blood samples are more difficult to compare and analyze, because of their small number, diverse designs and unknown miRNAs origin in the blood. Nevertheless, substantial variations in the whole blood c-miRNAs patterns between endurance and strength athletes and various exercise protocols were reported and support the idea that c-miRNAs are well suited to monitor the overall status of an athlete and the adaptation of organism to exercise [117,118,119,120,121,122,123,124,125].

Presented results of overviewed studies show that circulating miRNAs may distinguish between specific stress signals imposed by variations in the modality, duration and type of exercise.

## 7. Conclusions

The amount of research on miRNAs has grown exceedingly in last years. Many studies have proven a key role of miRNAs in the regulation of physiological adaptation to exercise, such as skeletal muscle and cardiomyocyte hypertrophy, mitochondrial biogenesis, vascular angiogenesis and metabolic processes. Exposure to exercise stimuli induces cellular activation leading to the physiological stress and tissue injury followed by repair and recovery. The summarized results of overviewed studies demonstrate that numerous tissue-specific miRNAs are released into circulation during and after the exercise and reflect the acute response to physiological stimulus. The c-miRNA expression pattern seems to be sensitive and specific for the type and intensity of exercise. Conventional biochemical biomarkers may be useful markers of tissue stress and injury, but they provide only limited information about the cellular mechanism of adaptation to the exercise. Circulating miRNAs meet most of the requirements for good biomarkers, such as minimally invasive and easily accessible sample collection, and remarkable stability in body fluids. 

However, there are many challenges associated with the detection of circulating miRNAs that need to be addressed. The lack of a standard protocol for the quantification of circulating miRNAs limits the cross-comparison of miRNA expression profiles between different methodologies and laboratories. Detection methods need standardization to minimize protocol-based bias, and the strategy of raw data normalization is a critical issue. To establish c-miRNAs as novel biomarkers, a key issue is to clarify their source and their relationship with the tissues or cells of origin. The possible correlation of c-miRNAs regulated by exercise with disease-modified c-miRNA patterns should be also taken into account during developing c-miRNAs signatures as physical performance biomarkers.

In spite of problems in quantification and uncertainties in their origin and function, c-miRNAs as blood-based biomarkers of exercise response are highly promising. Identification of c-miRNAs signatures characterizing a particular exercise modality may substantially impact the optimization of training, injury prevention and health status monitoring. However, well-designed and large-scale prospective studies through the examination of c-miRNAs across diverse populations which are exposed to exercise stimuli of variable types, duration and intensity, are required to validate the applicability of c-miRNA as biomarkers.

## Figures and Tables

**Table 1:** Circulating microRNAs (c-miRNA) plasma or serum profiles altered by various types of exercise in overviewed studies.

Study/Ref.	Exercise Type	Source	Detection	Altered Circulating miRNAs	Time Points
Baggish et al. 2011/[90]	Acute cycle ergometry test before sustained training	plasma	qPCR	↑ miR-21, -146a, -221, -222	Immediately after (decreased after 1 h)
	Sustained rowing training 90 days			↑ miR-20a, 21, -146a, -221, -222	At rest after sustained training
	Acute cycle ergometry test after sustained training			↑ miR-146a, -222	Immediately after
Uhlemann et al. 2014/[101]	Single symptom-limited spiroergometry test	plasma	qPCR	↑ miR-126	5 min. after finishing
	Cycling 4 h at 70% of anaerobic threshold			↑ miR-126	Immediately after
	Marathon run			↑ miR-126, -133	Immediately after
	Eccentric resistance exercise			↑ miR-133	Immediately after
Aoi et al. 2013/[99]	Acute—cycle ergometry 60 min. at 70% VO_2max_	serum	qPCR	↓ miR-486	Immediately after
	Systematic—cycling at 70% VO_2max_ 3 × 30 min. per week for 4 weeks			↓ miR-486	At rest after training
Baggish et al. 2014/[91]	Marathon run	plasma	qPCR	↑ miR-1, -126, -133a, -134,-146a, -208a, -499-5p	Immediately after (decreased after 24 h)
Mooren et al. 2014/[92]	Marathon run	plasma	qPCR	↑ miR-1, -133a, -206, -208b, -499	Immediately after
Gomes et al. 2014/[93]	Marathon run	plasma	qPCR	↑ miR-1, -133a, -206	Immediately after
De Gonzalo-Calvo et al. 2015/[94]	Marathon run	serum	qPCR panel of 106 inflammatory miRNAs	↑ let-7d-3p, let-7f-2-3p ↑ miR-29a-3p, -34a-5p, -125b-5p ↑ miR-132-3p, -143-3p, ↑ miR-148a-3p, -223-3p, -223-5p ↑ miR-424-3p, -424-5p	Immediately after (decreased after 24 h)
Clauss et al. 2016/[95]	Marathon run	plasma	qPCR	↑ miR-1, -30a, -133a	Immediately after (decreased after 24 h)
				↓ miR-26a, -29b	Immediately after
Min et al. 2016/[96]	Marathon run	plasma	qPCR	↑ miR-1, -133a, -206	Immediately after (decreased after 24 h)
Nielsen et al. 2014/[97]	Acute cycle ergometry test at 65% P_max_	plasma	qPCR 742 miRNAs panel	↓miR-30b, -106a, -146, -221, -652 ↓miR-151-3p, -151-5p, let-7i	Immediately after
				↑ miR-1, -133a, -133b, -139-5p ↑ miR-143, -145, -223, -330-3p, ↑ miR-338-3p, -223, -424	1–3 h post exercise
	Systematic endurance cycle ergometry training, 12 weeks			↑ miR-103, -107 ↓ miR-21, -25, -29b, -92a, ↓ miR-133a, -148a, -148b, ↓ miR-185, -342-3p, -766, let-7d	3–5 days after training
Cui et al. 2016/[100]	High intensity interval exercise	plasma	qPCR	**↑** miR-1, -133a, -133b, -206 **↑** miR-485-5p, -509-5p,-517a **↑** miR-518f, -520f, -522, -553 **↑** miR-888	Immediately after
	Vigorous-intensity continuous exercise			**↑** miR-1, -133a, -133b, -206 **↑** miR-485-5p, -509-5p,-517a **↑** miR-518f, -520f, -522, -553 **↑** miR-888	Immediately after
Banzet et al. 2013/[102]	Uphill treadmill test (concentric)	plasma	qPCR	**↑** miR-181b, -214	Immediately after
	Downhill treadmill test (eccentric)			**↑** miR-1, -133a, -133b, -208b	2–6 h after exercise
Sawada et al. 2013/[103]	Acute resistance exercise (bench press, leg press)	serum	Microarray qPCR	**↑** miR-149*↓ miR-146a, -221	3 days after exercise
Zhang et al. 2015/[104]	Systematic resistance training, 5 months	plasma	qPCR	**↑**miR-1, -133a, -133b, -206, -499, -208b	36–72 h after training
Wardle et al. 2015/[105]	Endurance training, 13 weeks	plasma	qPCR	**↑** miR-21, -221, -222, -146a (relative to control group)	At least 12 h post exercise
	Strength training, 13 weeks			↓ miR-21, -221, -222, -146a (relative to control group)	

**Table legend:** **↑**—upregulated miRNAs; ↓—downregulated miRNAs.

**Table 2:** circulating microRNAs (c-miRNAs) with differential expression profiles in plasma or serum depending on type of exercise and sample collection.

c-miRNA	Regulation	Exercise Type	Time Points	Ref.
miR-21	up	Endurance acute	Immediately after	[90]
	up	Endurance chronic	At rest after	[90]
	up	Endurance chronic	At least 12 h after	[105]
	down	Strength chronic	At least 12 h after	[105]
	down	Endurance chronic	3–5 days after	[97]
miR-146a	up	Endurance acute	Immediately after	[90]
	up	Endurance chronic	At rest after	[90]
	up	Endurance acute (M)	Immediately after	[90]
	up	Endurance chronic	At least 12 h after	[105]
	down	Strength chronic	At least 12 h after	[105]
	down	Endurance acute	Immediately after	[97]
	down	Strength acute	3 days after	[103]
miR-148a	up	Endurance acute (M)	Immediately after	[94]
	down	Endurance chronic	3–5 days after	[97]
miR-221	up	Endurance acute	Immediately after	[90]
	up	Endurance chronic	At rest after	[90]
	up	Endurance chronic	At least 12 h after	[105]
	down	Strength chronic	At least 12 h after	[105]
	down	Endurance acute	Immediately after	[97]
	down	Strength acute	3 days after	[103]
miR-222	up	Endurance acute	Immediately after	[90]
	up	Endurance chronic	At rest after	[90]
	up	Endurance chronic	At least 12 h after	[105]
	down	Strength chronic	At least 12 h after	[105]
Let-7d	up	Endurance acute (M)	Immediately after	[94]
	down	Endurance chronic	3–5 days after	[97]

Up—upregulated; down—downregulated; (M)—marathon run.
